# Antimicrobial Photoinactivation Using Visible Light Plus Water-Filtered Infrared-A (VIS + wIRA) Alters *In Situ* Oral Biofilms

**DOI:** 10.1371/journal.pone.0132107

**Published:** 2015-07-10

**Authors:** A. Al-Ahmad, M. Bucher, A. C. Anderson, C. Tennert, E. Hellwig, A. Wittmer, K. Vach, L. Karygianni

**Affiliations:** 1 Department of Operative Dentistry and Periodontology, Center for Dental Medicine, Albert-Ludwigs-University, Freiburg, Germany; 2 Institute of Medical Microbiology and Hygiene, Albert-Ludwigs-University, Freiburg, Germany; 3 Institute for Medical Biometry and Statistics, Center for Medical Biometry and Medical Informatics, Albert-Ludwigs-University, Freiburg, Germany; Massachusetts General Hospital, UNITED STATES

## Abstract

Recently, growing attention has been paid to antimicrobial photodynamic therapy (aPDT) in dentistry. Changing the microbial composition of initial and mature oral biofilm by aPDT using visible light plus water-filtered infrared-A wavelengths (VIS + wIRA) has not yet been investigated. Moreover, most aPDT studies have been conducted on planktonic bacterial cultures. Therefore, in the present clinical study we cultivated initial and mature oral biofilms in six healthy volunteers for 2 hours or 3 days, respectively. The biofilms were treated with aPDT using VIS+wIRA (200 mW cm^-2^), toluidine blue (TB) and chlorine e6 (Ce6) for 5 minutes. Chlorhexidine treated biofilm samples served as positive controls, while untreated biofilms served as negative controls. After aPDT treatment the colony forming units (CFU) of the biofilm samples were quantified, and the surviving bacteria were isolated in pure cultures and identified using MALDI-TOF, biochemical tests and 16S rDNA-sequencing. aPDT killed more than 99.9% of the initial viable bacterial count and 95% of the mature oral biofilm *in situ*, independent of the photosensitizer. The number of surviving bacterial species was highly reduced to 6 (TB) and 4 (Ce6) in the treated initial oral biofilm compared to the 20 different species of the untreated biofilm. The proportions of surviving bacterial species were also changed after TB- and Ce6-mediated aPDT of the mature oral biofilm, resulting in a shift in the microbial composition of the treated biofilm compared to that of the control biofilm. In conclusion, aPDT using VIS + wIRA showed a remarkable potential to eradicate both initial and mature oral biofilms, and also to markedly alter the remaining biofilm. This encourages the clinical use of aPDT with VIS + wIRA for the treatment of periimplantitis and periodontitis.

## Introduction

In recent years, the application of photodynamic therapy (PDT) as an antimicrobial treatment approach has gained in importance [1–4]. The rising number of antibiotic-resistant microorganisms in different fields of medicine justifies the increasing focus on PDT as an alternative method to treat infections [2,3]. Microbial biofilms are considered to be the cause of 60–80% of infections in medicine [5]. Moreover, microorganisms living in biofilms are up to 1000 times more resistant against antimicrobials than their planktonic counterparts [6]. Resistance mechanisms of microorganisms within biofilms include the slow penetration of antimicrobials within the biofilms, and also involve the response and alteration of the growth rate and the microenvironment within the biofilm [6–8]. Due to the mode of action of PDT, which inactivates major metabolic pathways and structures of the microbial cells, the development of resistance against this treatment modality can be excluded, making it a promising approach to overcome microbial resistance mechanisms against antimicrobials [9].

Although oral biofilm is the etiological base associated with caries and periodontitis, the most prevalent dental diseases in industrialized countries, only two studies used native *in situ* dental plaque to study the effects of antimicrobial PDT (aPDT) to eradicate biofilm bacteria and to evaluate aPDT as an alternative approach to treat oral biofilms [3,4]. In their recent review Cieplik *et al*. [10] summarized studies about the inactivation of oral biofilms formed by key oral pathogens and showed that only single species *in vitro* biofilms were studied. This fact underlines the need for studying the effects of aPDT on the highly diverse *in situ* initial and mature biofilm which is formed within the realistic and complex conditions of the oral cavity, not only on the natural enamel tooth surface, but on those of different dental implants as well [11–14].

Until now, the effects of aPDT in combination with light-emitting-diode (LED) and wide-band halogen lamps as light sources has been intensely investigated in various studies of planktonic bacterial cultures, as has been summarized in a recent systematic review [10]. However, low-priced LED appliances have a restricted emission wavelength spectrum, and the wide-band halogen lamps which were used can induce tissue overheating [15]. Therefore, the development of a broad-band light source consisting of visible light (VIS) wavelengths in combination with water-filtered infrared-A (wIRA) wavelengths has been shown to be a promising alternative source of light [4]. The combination of VIS and wIRA was also reported to increase oxygen partial pressure in tissue, leading to higher *in situ* temperature and perfusion levels, in turn inducing chronic wound healing and a reduction in pain [16]. Furthermore, due to its significant subcutaneous tissue penetration, wIRA protects external tissue layers by decreasing the immense thermal stress [17,18]. Compared to other sources the main advantages of VIS + wIRA are not only its thermal and thermic effects but also its non-thermal and non-thermic effects [19]. The main thermal response of tissues to wIRA includes a mild increase in subcutaneous temperature, higher tissue oxygen partial pressure and perfusion levels, which lead to chronic wound healing. Interestingly, it was found that in a tissue depth of 2 cm, the subcutaneous temperature increased only by 2.7°C [20]. On the other hand, LED induced a higher temperature increase of up to 7°C in the tooth pulp chamber [21]. LED appliances have a low cost but their emission wavelength spectrum is rather limited.

In recent studies, we showed high antimicrobial effects of aPDT using VIS + wIRA in combination with the photosensitizers toluidine blue (TB) and chlorine e6 (Ce6) against initial and mature *in situ* oral biofilm [3,4]. Using aPDT with VIS + wIRA in combination with Ce6 and TB has been shown to be effective for the eradication of planktonic *Streptococcus mutans* and *Enterococcus faecalis* as well as the initial oral biofilm [3]. Furthermore, aPDT with VIS +wIRA and the aforementioned photosensitizers was able to significantly kill mature oral biofilms cultured *in situ* [4]. To date, the effects of aPDT on the microbial diversity of surviving biofilm bacteria have not been studied. These encouraging results together with the health benefits of this technique mentioned above led us to the assumption that aPDT in combination with VIS and wIRA could be a promising alternative method for the treatment of periodontitis and periimplantitis, both of which are diseases caused by oral biofilms. Keeping in mind that these oral diseases correlate with pathological shifts in the biofilms of the supragingival and subgingival dental plaque [22,23], alteration of oral biofilm composition during treatment could influence the healing and eradication process. Nevertheless, changing the ecological balance of the oral biofilm through the use of aPDT has not been studied to date. This would augment the efficiency of aPDT, due to the fact that antimicrobial properties of aPDT can be increased in the presence of endogenous photosensitizers, as has been previously revealed for the key periodontal pathogen *Aggregatibacter actinomycetemcomitans* [24]. The aim of the present study was to analyze the surviving microorganisms after applying a novel aPDT approach using VIS + wIRA in combination with TB and Ce6 as photosensitizers. For this purpose, intact oral biofilms grown on bovine enamel slabs (BES) *in vivo* within the oral cavity for 2 hours (h) or 3 days (d), respectively, were treated photodynamically using VIS + wIRA. The surviving bacteria from the treated initial and mature oral biofilms were determined, and the isolated species were identified. To the best of our knowledge, this is the first time that the effect of VIS + wIRA combined with TB or Ce6 has been tested on *in situ* initial and mature oral biofilms to study their effects on the composition of oral biofilm.

## Materials and Methods

### Selection of study participants and test specimens

Six healthy volunteers (5 females, 1 male) between 25 and 54 years of age participated in the study. The study protocol was reviewed and approved by the Ethics Committee of the University of Freiburg (Nr. 91/13). All volunteers gave their written informed consent prior to the start of the study. A thorough clinical oral examination was conducted prior to the start of the experiments. The following were used as exclusion criteria for the study: 1) severe systemic disease, 2) diseases of the salivary glands, 3) presence of carious lesions or periodontal disease, 4) pregnancy or lactation, 5) use of antibiotics or local antimicrobial mouth rinses such as chlorhexidine (CHX) within the last 30 days. All participants were non-smokers. DMFT values (decayed, missing, filled teeth) of 4.5 ± 3 were measured, salivary flow rates were estimated at 1.2 ± 0.3 ml / min, while the lactate formation rates were at 2.5 ± 0.6 (scale from 1 to 9) [2,4]. These values underlined the healthy oral status of the volunteers involved in this study.

In order to prepare the test specimens, bovine incisors of freshly slaughtered 2-year old BSE (spongiform encephalopathy)-free cattle were used. The cattle were slaughtered in the Slaughterhouse of Freiburg (Freiburg, Germany). Cylindrical enamel samples (diameter 5 mm, 19.63 mm^2^ surface area, height 1 mm) were prepared as has been described previously [14,25]. Afterwards, the enamel surfaces of all specimens were polished by a wet grinding machine (Knuth-Rotor-3, Streuers, Willich, Germany) using wet sandpaper (abrasive grading scales from 250 to 4000 grit) in decreasing order of grain size, as has been described recently [14,25]. The surface of prepared bovine enamel slabs (BES) were then controlled under a light microscope (Wild M3Z, Leica GmbH, Wetzlar, Germany). The BES were disinfected by ultrasonication in NaOCl (3%) for 3 min to remove the superficial smear layer, followed by air drying and ultrasonication in 70% ethanol for a further 3 min. To remove residues of NaOCl and ethanol the disinfected BES were then ultrasonicated twice in double distilled water for 10 min. Prior to use the BES were stored in sterile distilled water for at least 24 h in order for them to hydrate before fixing them in the acrylic appliances described below which were then applied in the oral cavity [14,25].

**Fig 1:**
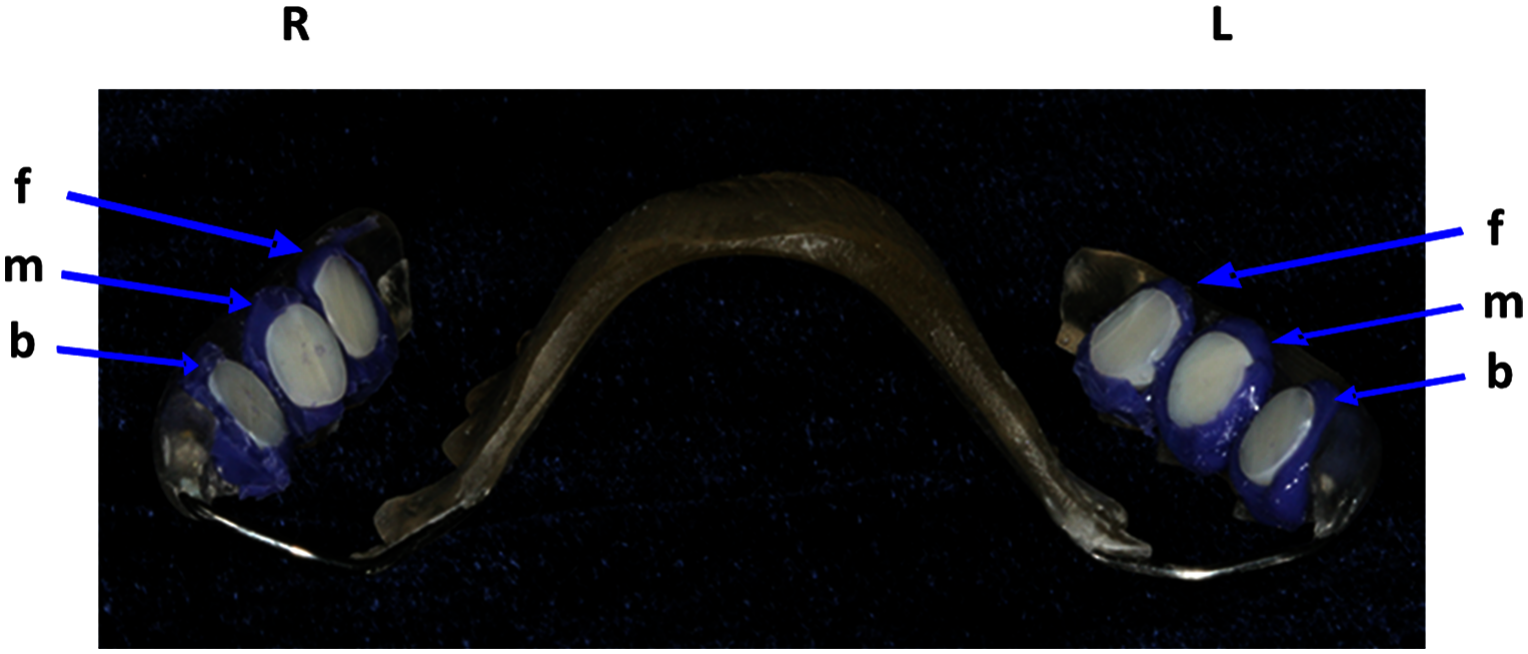
Individual upper jaw acrylic splint with six bovine enamel slabs (BES) embedded in silicon at different sites. The BES were fixed at the front (f), in the middle (m) and at the back (b), for both the right (R) and left (L) sides of the splint. Silicon covered the downward-facing surfaces and the margins of the BES and left only their upward-facing surfaces exposed.

Individual upper jaw acrylic appliances were prepared for each study volunteer and six BES were fixed on their approximal sites using an A-silicon compound (Panasil initial contact X-Light, Kettenbach GmbH & Co. KG, Eschenburg, Germany), as has been described elsewhere [14,25]. To ensure that only the BES surfaces were exposed to the oral cavity, their margins were fully covered by the impression material (Fig 1). The BES were subsequently fixed to the interdental area between upper premolars and molars. Subsequently, the biofilm formation was not disturbed by the movements of the tongue or cheek for either 2 h or 3 d, respectively. A total of twelve BES were carried by every volunteer for each time period of biofilm formation. To provide a sufficient number of biofilm samples for the aPDT assays, each participant wore the BES-incorporating acrylic appliance twice for each time period. During the 3-day experiments the volunteers brushed their teeth. During performance of oral hygiene and the meals the BES-containing splints were deposited in 0.9% saline solution and they were not brushed during this time. The splints were not removed during sleep.

### Light source and photosensitizers

The biofilm samples which gained *in situ* were then treated using a broad-band VIS + wIRA radiator (Hydrosun 750 FS, Hydrosun Medizintechnik GmbH, Müllheim, Germany) with a 7 mm water cuvette as has been described previously [3,4]. The principle of operation involves the use of the hermetically sealed water filter in the radiation path to absorb the infrared-B and-C wavelengths emitted by a halogen lamp (dimensions: length: 28 cm, width: 27 cm, height: 28 cm) with a power input of 750 W (rated voltage: 230 V, 50–60 Hz). Additionally, two distinct absorption bands at 944 and 1180 nm were also filtered in order to minimize superficial overheating and exsiccosis. The distance between the biofilm samples and the light source was 20 cm. An accessory orange filter, BTE31, was adapted to the light source, which had a diameter of 10 cm and an output power of 200 mW / cm^2^. Due to the absorption of water molecules, the continuous water-filtered spectrum covered a wavelength range from 570 nm to 1400 nm, with local minima at 970 nm, 1200 nm and 1430 nm, respectively [26]. The unweighted (absolute) total irradiance applied to the biofilm samples for 5 min amounted to 200 mW cm^-2^ VIS + wIRA, which consisted of approximately 48 mW cm^-2^ VIS and 152 mW cm^-2^ wIRA.

The VIS + wIRA broadband light source used in this study allowed for optimal light absorption by the chosen photosensitizers. The photosensitizers used were toluidine blue O (TB) (C_15_H_16_ClN_3_S, Sigma-Aldrich, Munich, Germany) and chlorine e6 (Ce6) (C_34_H_36_N_4_O_6_, Frontier Scientific, Logan, UT, USA). TB and Ce6 solutions were prepared in 0.9% saline (NaCl) to a final concentration of 100 μg ml^-1^. Prior to use, the TB and Ce6 solutions were stored in the dark at 4°C for no longer than 14 days to prevent any light-induced photochemical attenuation. The optical absorption spectrum of TB extended from 500 nm to 700 nm. The visible absorption maximum (λ max) of TB was 630 ± 4 nm; additional λ maxima of TB were also measured at 570 nm and 650 nm [27]. The optical absorption spectrum of Ce6 revealed maximum absorption peaks at 403 ± 2 nm (Soret band) and 664 ± 3 nm (Q band), respectively [28].

### aPDT protocol for oral biofilms

**Fig 2:**
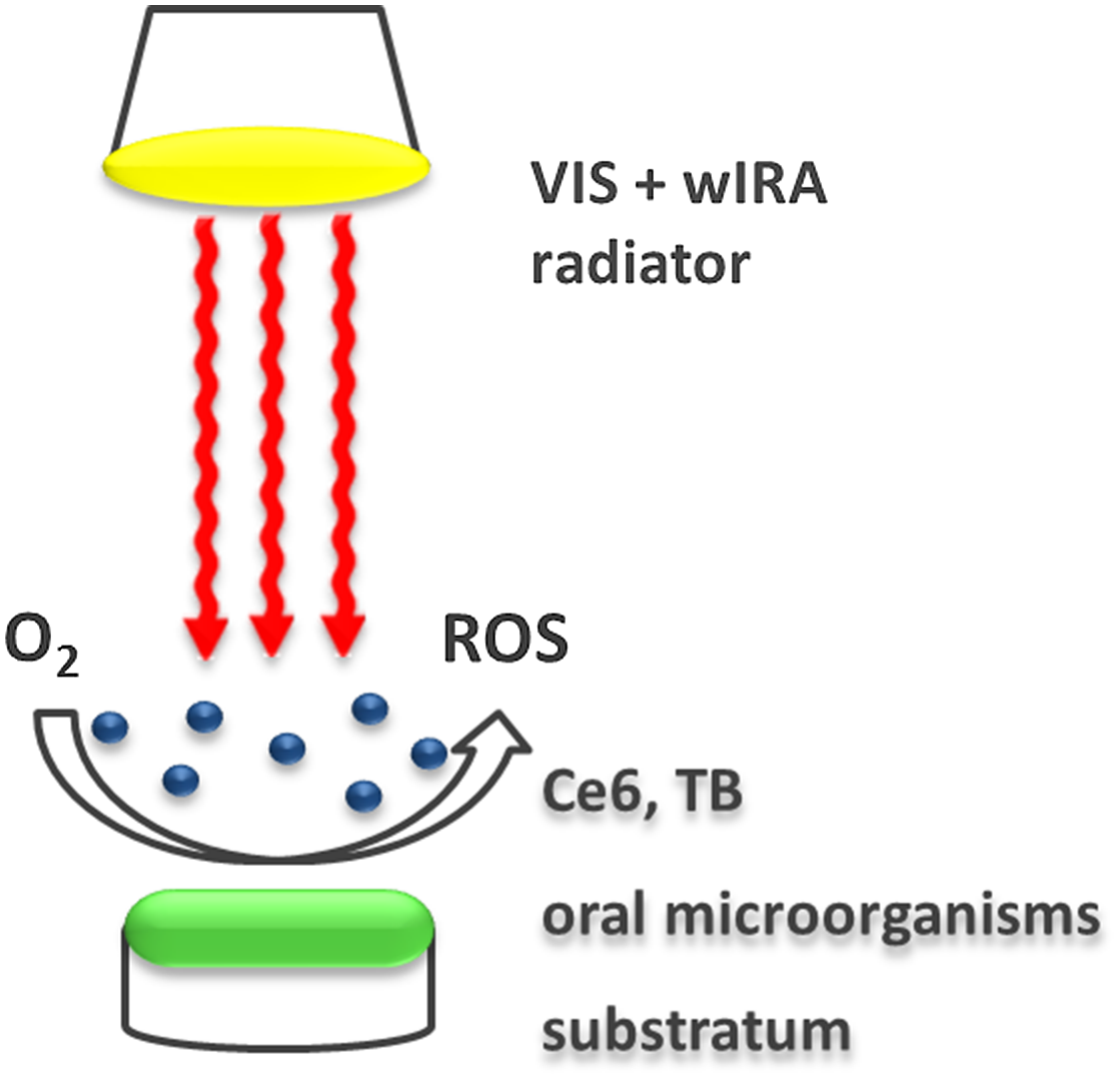
Representative illustration of the application of antimicrobial photodynamic therapy using visible light (VIS) plus water-filtered infrared-A (wIRA) on oral microorganisms. In brief, a VIS + wIRA radiating device with a broadband water-filtered spectrum (570–1400 nm) enables the excitation of the tested photosensitizers toluidine blue (TB) and chlorine e6 (Ce6). As a result, these interact with oxygen (O_2_) inducing the release of a variety of reactive oxygen species (ROS), which can destroy planktonic and oral biofilm microorganisms.

For initial and mature biofilm formation, each volunteer carried an individual upper jaw acrylic appliance containing six bovine enamel samples (BES) for 2 h or 3 d, respectively. This procedure was performed twice for each subject and time period. After the oral biofilm formation period *in situ*, the acrylic appliance was removed from the oral cavity. The BES covered with biofilm were removed from the splint using sterile tweezers. The BES were then immediately rinsed off with sterile 0.9% NaCl for 30 s to remove unattached bacterial cells. One specimen out of a total of six BES per participant was treated with 0.2% CHX and served as a positive control, while one untreated specimen was used as a negative control. Two biofilm-covered BES were treated with aPDT *ex vivo* using VIS + wIRA and TB at a concentration of 100 μg ml^-1^. Two additional BES were treated with aPDT and 100 μg ml^-1^ Ce6. For aPDT treatment, the BES were placed into multiwell plates (24-well plate, Greiner bio-one GmbH, Frickenhausen, Germany) and incubated with the photosensitizers for 2 min in the dark, in duplicate. Then, the VIS + wIRA radiation was applied for 5 min at 37°C (Fig 2). The specimen used as positive control was incubated for 5 minutes in 0.2% CHX. For the determination of CFU it was washed with 0.9% saline solution and transferred to 1 ml 0.9% saline. The negative control was incubated in 100 μl 0.9% saline for 5 minutes prior to the quantification of the adhered oral biofilm microorganisms. Immediately after the aPDT the quantification and isolation of adherent microorganisms was conducted as described below. After aPDT treatment the BES were transferred into multiwell plates with 1 ml 0.9% NaCl and adherent microorganisms were finally quantified based on the colony forming units (CFU) and identified using different methods as described in the following text.

### Quantification of the adhered oral biofilm microorganisms

To remove non-adherent microorganisms, the margins and the bottom dentine surfaces of the biofilm-covered bovine enamel samples (BES) were brushed down using small sterile foam pellets (Voco GmbH, Cuxhaven, Germany). The BES were then washed with 1 ml 0.9% NaCl for 10 s to dislodge residual non-adherent microorganisms. Afterwards, the BES were inserted into sterile Eppendorf tubes (Eppendorf GmbH, Wesseling-Berzdorf, Germany) with 1 ml 0.9% NaCl and ultrasonicated for 2 min in 1 ml NaCl on ice. They were then finally vortexed for 30–45 s to release the adherent microorganisms of the initial and mature oral biofilm from the surface. The suspensions of treated BES, untreated BES (negative control) and CHX-treated BES were serially diluted up to 1:10^3^ (initial oral biofilm) and up to 1:10^5^ (mature oral biofilm) in 0.9% NaCl. Different dilutions were plated taking the detection limit of CFU on nonselective media into consideration. This detection limit could vary dependent on the tested bacterial species (usually in the range of 10-10^3^ CFU per ml of the detached biofilm bacteria). Subsequently, the bacteria were cultured and identified as described elsewhere [29]. In brief, to isolate and identify the microorganisms 100 μl of each dilution were plated on yeast-cysteine blood agar plates (HCB) and on Columbia blood agar plates (CBA). HCB agar plates were used to cultivate anaerobic bacteria at 37°C for 10 days (anaerobic chamber, GENbox BioMérieux sa, Marcy l’Etoile-France). Aerobic and facultative anaerobic bacteria were cultivated and isolated on CBA agar plates after incubation at 37°C and 5%-10% CO_2_ atmosphere for 5 days. The colony forming units (CFU) were counted and calculated per ml in the original sample. All colony types were noted and sub-cultivated to obtain pure cultures.

Gram stains were prepared and bacterial cell morphology was determined using light microscopy (Axioscope; Zeiss, Jena, Germany; 1000X magnification). The identification of the pure bacterial cultures was conducted using MALDI-TOF analysis in a MALDI Biotyper Microflex LT (Maldi Biotyper, Bruker Daltonik GmbH, Bremen, Germany) as described elsewhere [30]. In brief, bacteria from single pure colonies were used for MALDI-TOF analysis. The mass spectra were acquired according to the manufacturer’s recommendations. The BioTyper 3.0 software compared the obtained spectra with a reference database containing 3740 reference spectra (representing 319 genera and 1,946 species). The resulting similarity value was expressed as a log score. Identification at the species level was indicated by a score of ≥ 2.0, whereas a score of ≥ 1.7 indicated identification at the genus level. Any score under 1.7 meant no significant similarity of the obtained spectrum to any database entry. The procedure was repeated if the results were questionable. In addition, universal bacterial PCR was carried out using the following primers: TP16U1: 5’-AGAGTTTGATCMTGGCTCAG-3’ and RT16U6: 5’-ATTGTAGCACGTGTGTNCCCC-3’ followed by sequencing if the MALDI-TOF analysis was questionable. Sequencing was performed on a 3130 Genetic Analyzer (Applied Biosystems, Life Technologies GmbH, Darmstadt, Germany).

### Statistical Analysis

The results of killing effects of CFU were calculated together for all 6 participants to reveal the efficacy of aPDT in combination with the respective photosensitizer. The identification and quantification of the surviving oral microorganisms were analyzed both for each volunteer separately and for all volunteers together as well. The means and standard deviations were computed for a descriptive evaluation of the data. A Friedman test was conducted to check for overall differences between the test groups regarding the microbial load. For pair-wise group comparisons a t-test with Bonferroni correction (multiple testing) was used. Due to the small number of cases a non-parametric test has not enough power and a t-test is conservative. Viable bacterial counts on the log_10_ scale per square centimeter (log_10_ / cm^2^) were graphically depicted and stratified by biofilm age (initial / mature). All calculations were performed using the statistical software STATA 13.1.

## Results

### aPDT effects on the viable counts of oral microorganisms in initial adhesion and mature oral biofilms

**Fig 3:**
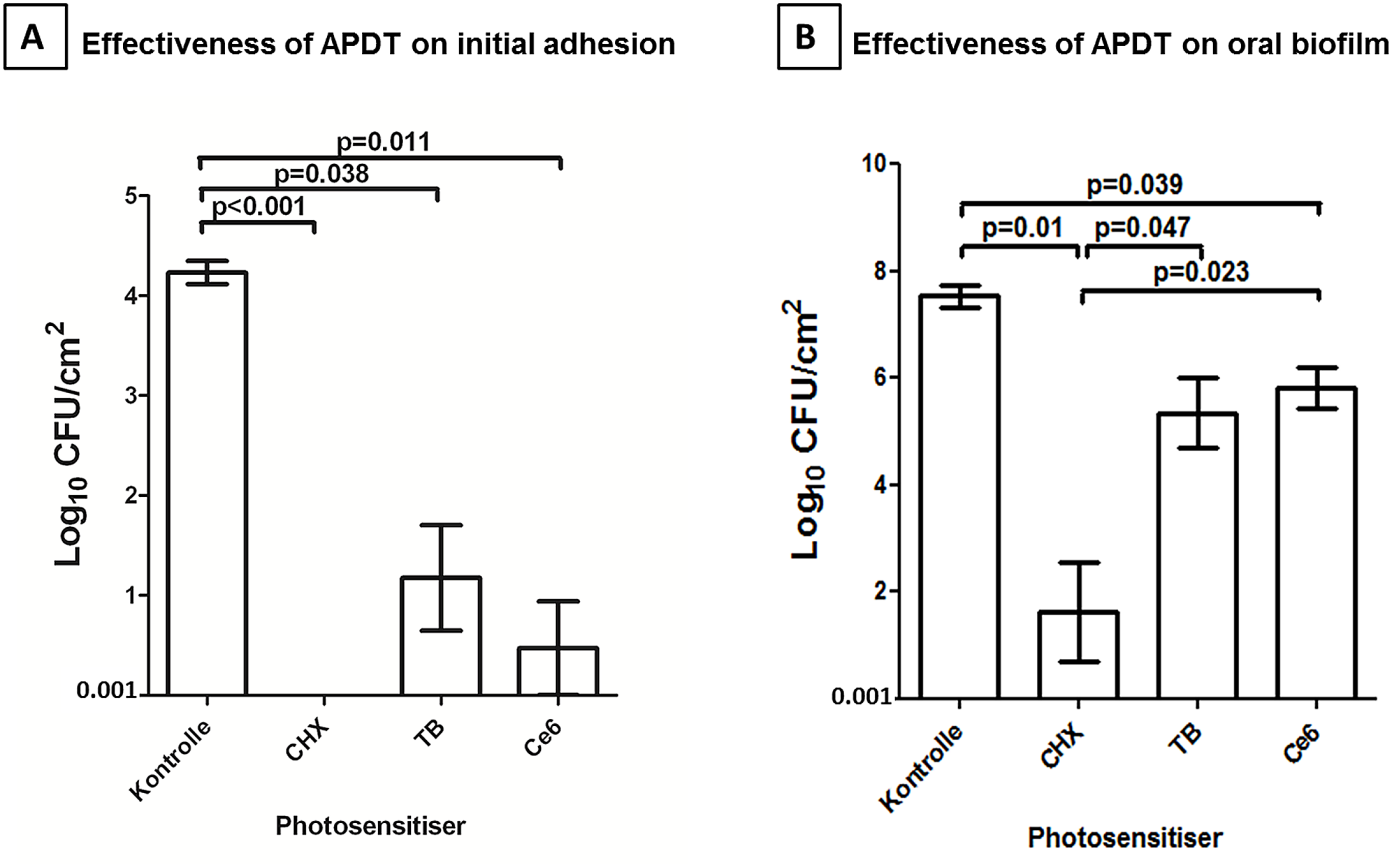
Diagram of the colony forming units (CFUs) depicting the effect of photodynamic therapy using visible light (VIS) plus water-filtered infrared-A (wIRA) against oral microorganisms during initial adhesion (A) and biofilm formation (B), respectively. The photosensitizers toluidine blue (TB) and chlorine e6 (Ce6) were applied (100 μg/ml) *ex vivo* onto the oral microorganisms after 2 hours (h) or 3 days (d) of *in situ* cultivation, respectively. An untreated specimen and a chlorhexidine-treated (CHX) specimen served as negative and positive controls. A log_10_ scale per square centimeter (log_10_ / cm^2^) indicates the CFUs. The p-values (t-test) of the significantly different data are depicted above.

Fig 3(A, B) shows the eradication rates of initially adherent oral bacteria in the initial oral biofilm (A) and in the mature three-day-old oral biofilm (B) after application of aPDT using VIS + wIRA in the presence of TB and Ce6, as well as for the untreated positive (CHX) and negative controls. aPDT killed more than 99.9% of the viable bacterial count of initial microbial adhesion *in situ*, independent of the photosensitizer used. The untreated control for initial microbial adhesion revealed a log_10_ CFU value of 4.23 ± 0.28, while the CFU counts of the CHX-treated positive control were completely killed (100%). Application of aPDT using TB showed a significant decrease (p = 0.038) of 3.06 CFU (mean 1.17 ± 1.29) on a log_10_ scale, while aPDT using Ce6 also resulted in a significant reduction (p = 0.011) of 3.76 log_10_ CFU (mean 0.47 ± 1.15).

Fig 3(B) demonstrates the killing rates of adherent microorganisms within the three-day-old oral biofilm after treatment with VIS + wIRA-derived aPDT using TB and Ce6 as photosensitizers, as well as for the CHX-treated positive control and the untreated negative controls. The treatment of the oral biofilm with aPDT in combination with either TB or Ce6 revealed significant decreases in the viable counts for oral biofilm microorganisms, corresponding to a minimum reduction of 95%. TB-mediated aPDT (mean 5.33 ± 1.61) showed a significant reduction in CFU (p = 0.024) of 2.21 log_10_ compared with the untreated negative control (mean 7.54 ± 0.50). The application of aPDT using Ce6 (mean 5.81.41 ± 0.92) significantly reduced (p = 0.016) CFU counts by 1.73 log_10_ compared to the negative control. CHX (mean 1.61 ± 2.26) greatly reduced the number of viable bacteria in the oral biofilm by 5.93 log10 (p = 0.004).

### Shift of bacterial spectrum after use of aPDT on initial microbial adhesion

**Fig 4:**
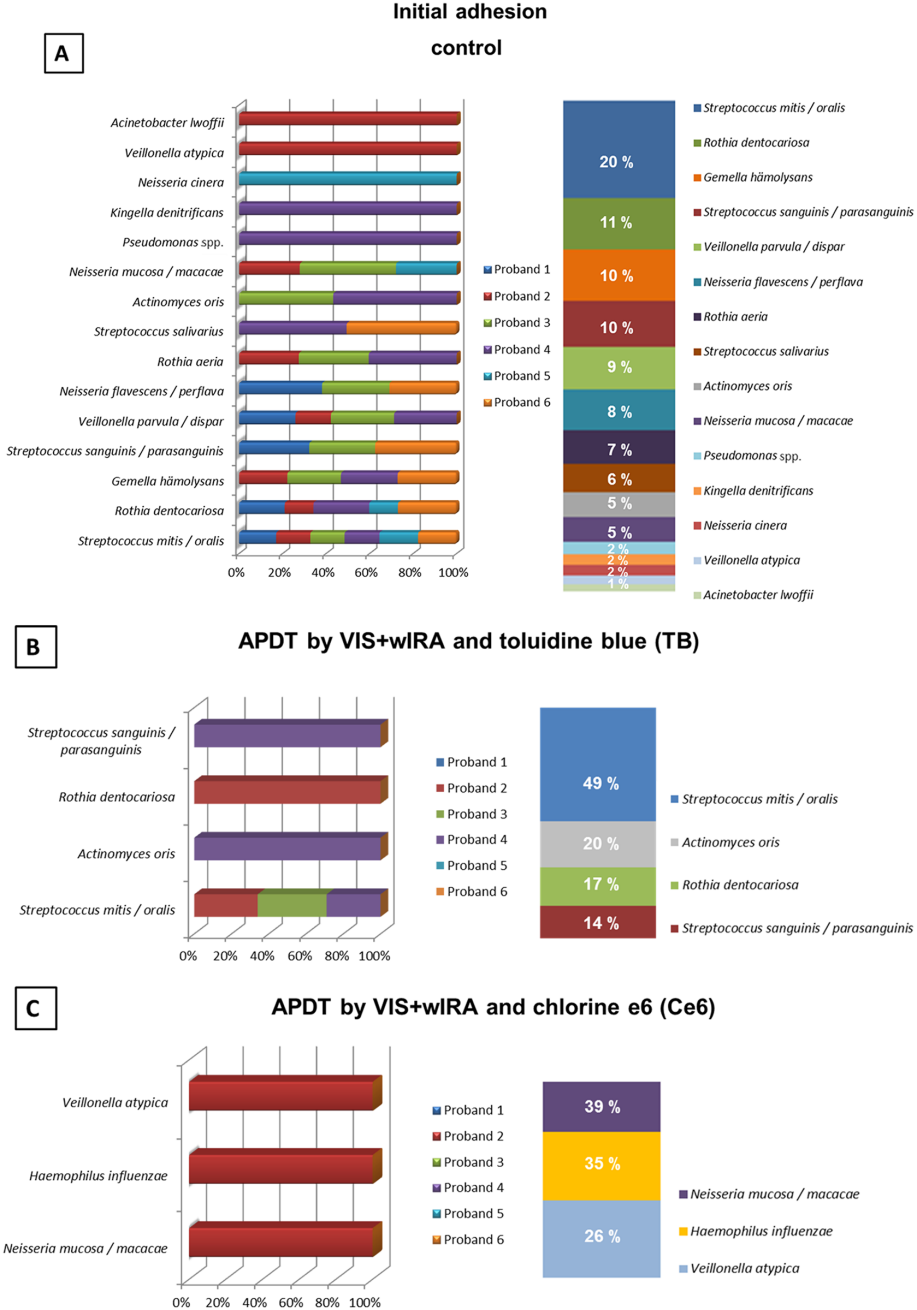
Relative distribution among the volunteers and total distribution (in percentages) of the initially adherent bacterial species from 6 individuals after application of antimicrobial photodynamic therapy (aPDT) using visible light (VIS) plus water-filtered infrared-A (wIRA). Graph A depicts the bacterial composition of the untreated negative controls. Graphs B and C show the bacterial composition after the *ex vivo* treatment with VIS + wIRA and the photosensitizers (100 μg/ml) toluidine blue (TB) and chlorine e6 (Ce6) on oral microorganisms after a 2-hour (h) cultivation *in situ*. Consistent color coding was used for the study participants and detected bacteria as indicated by the schemes at the left and right of panels A, B and C respectively.

The diversity of cultured bacteria in untreated initial adhesion samples were analyzed and compared with the diversity of surviving bacteria after aPDT treatment with toluidine blue (TB) and chlorine e6 (Ce6), respectively. After treatment with CHX (positive control) all bacteria were killed, leading to the elimination of the original bacterial community shown in Fig 4A for the untreated initial adhesion (negative control). It was possible to isolate and identify 19 different bacterial species. The total distribution in percentage of different bacterial species was between 20% for *Streptococcus mitis* / *oralis* and 1% for *Acinetobacter lwofii*. *Rothia dentocariosa*, *Gemella hämolysans* and *Streptococcus sanguinis* / *parasanguinis* were each found in amounts of 10–11% of the total isolated bacteria. The percentages of all other species among all 6 probands depicted in Fig 4A ranged from 2–9%. The most prevalent species were *S*. *mitis* / *oralis* (in all 6 volunteers) followed by *R*. *dentocariosa* (in 5 volunteers) and *G*. *hämolysans* and *Veillonella parvula* (4 volunteers, each). All other species were detected in 3 or less volunteers.

As shown in Fig 4B, the aPDT with VIS+wIRA and TB reduced the number of different surviving bacterial species from 19 to 6, including *S*. *mitis* / *oralis* (49% total distribution), *Actinomyces oris* (20%), *R*. *dentocariosa* (17%) and *S*. *sanguinis* / *parasanguinis* (14%). The total distribution among the probands was highly influenced: No bacteria were cultured in three volunteers, whereas *S*. *mitis* / oralis were detected in only three volunteers and each of the other species were only detectable in a single volunteer. The aPDT using VIS+wIRA and Ce6 (Fig 4C) resulted in a high reduction of species diversity from 19 to 4 species, which were detected in only one volunteer who also showed a reduction in the diversity of the surviving bacteria.

### Shift of bacterial community composition after aPDT application to the mature oral biofilm

**Fig 5:**
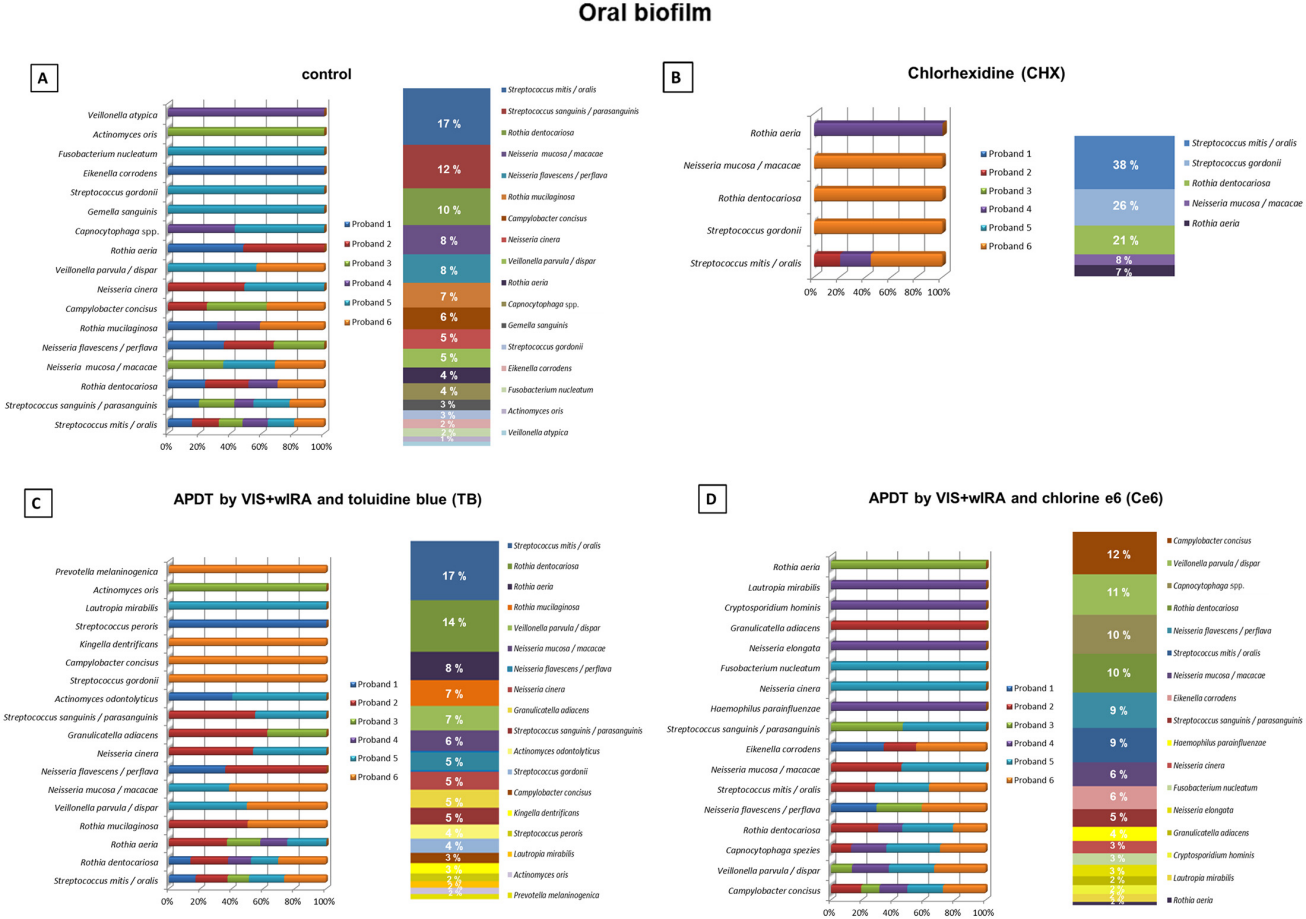
Relative distribution among volunteers and total distribution in percentages of oral biofilm bacterial species from 6 individuals after the application of antimicrobial photodynamic therapy (aPDT) using visible light (VIS) plus water-filtered infrared-A (wIRA). Graph A depicts bacterial composition of the untreated negative controls. Graph B demonstrates bacterial composition of the 0.2% chlorhexidine (CHX)-treated positive controls. Graphs C and D show the bacterial composition after the *ex vivo* treatment with VIS + wIRA and the photosensitizers (100 μg/ml) toluidine blue (TB) and chlorine e6 (Ce6) after 3-day (d) cultivation of the oral microorganisms *in situ*. Consistent color coding was used for the study participants and detected bacteria as indicated by the schemes at the left and right of panels A, B, C and D respectively.

The relative distribution of the surviving bacterial species after treatment of the mature oral biofilms with aPDT using toluidine blue (TB) and chlorine e6 (Ce6) in percentage and the total distribution among all volunteers are shown in Fig 5C and 5D. The corresponding results for the untreated negative controls and the positive controls treated with CHX are shown in Fig 5A and 5B, respectively. CHX highly affected the bacterial diversity of the biofilms, reducing the number of the detected different species which survived the treatment from 22 to 7. These species were found primarily in one volunteer. *Streptococcus mitis* / *oralis* were detected in three different volunteers after treatment with CHX.

Compared to the negative control, application of aPDT in combination with TB increased the percentage of *Rothia dentocariosa*, *Rothia aeria*, *Veillonella parvula* / *dispar* and *Streptococcus gordonii*. On the other hand, the portions of *Neisseria mucosa* / *macacae*, *Neisseria flavescens* / *perflava*, *Streptococcus sanguinis* / *parasanguinis* and *Campylobacter concisus* were decreased. *Capnocytophaga* spp., *Gemella sanguinis*, *Eikenella corrodens*, *Fusobacterium nucleatum* and *Veillonella atypica* were not detectable after TB-mediated aPDT treatment. This means that 13% of all bacterial species isolated in the negative control were totally eliminated by aPDT treatment. Interestingly, six different species (19% of all surviving bacteria) including *Granulicatella adiacens*, *Actinomyces odontolyticus*, *Kingella dentrificans*, *Streptococcus peroris*, *Lautropia mirabilis* and *Prevotella melaninogenica* could only be detected after the aPDT treatment using TB. As a consequence, the total distribution of the detected species were also changed as depicted in Fig 5C.

Ce6 mediated aPDT also caused a shift in the diversity of surviving bacteria. The relative proportions of 7 species (*Campylobacter concisus*, *Veillonella parvula* / *dispar*, *Capnocytophaga* spp., *Neisseria flavescens* / *perflava* and *Eikenella corrodens*) increased, whereas the percentages of 7 species (*S*. *mitis* / *oralis*, *N*. *mucosa* / *macacae*, *S*. *sanguinis* / *parasanguinis* and *N*. *cinera*) decreased. Five species (16% of all bacterial species detected in the untreated control), including *R*. *mucilaginosa*, *G*. *sanguinis*, *S*. *gordonii*, *Actinomyces oris* and *V*. *atypica*, could not be detected after aPDT treatment using Ce6. In contrast, 5 species which were not detected in the control were revealed in the aPDT treated biofilm. Accordingly, the total distribution among the volunteers was also changed as depicted in Fig 5D.

## Discussion

Antimicrobial photodynamic therapy (aPDT) has recently become an interesting alternative approach in several medical fields, including dentistry [3,4,10,31]. However, most studies were conducted to investigate the effects of aPDT on planktonic bacteria [10]. To date, some clinical studies have confirmed the favorable effects of aPDT using various light sources on multispecies oral biofilms of periodontitis patients [32–34]. Only two studies exist describing the effects of aPDT using visible light plus water-filtered infrared-A (VIS + wIRA) as a novel light source [3,4], while only one study dealt with mature oral biofilm [4]. Independent of the light sources, photosensitizers and microbial targets, all reports existing on aPDT to date have only described the killing effects on microorganisms and the reduction of the microbial load targeted with aPDT. This approach is adequate if a single planktonic bacterial species or a single-species biofilm is treated by aPDT. In contrast, if a highly diverse microbial niche is targeted with aPDT, the influence of this particular technique on the diversity of surviving microorganisms is a major concern, in addition to the total reduction in the microbial count. This point has been overlooked until now, when the potential of aPDT for eradication of the oral biofilm was discussed. The present study for the first time assesses the effects of aPDT on the diversity of surviving bacteria in initial and mature oral biofilms cultivated *in situ*. Besides the innovation of using VIS + wIRA as a light source, a diverse natural microbial niche was targeted using the two different photosensitizing agents toluindie blue (TB) and chlorine e6 (Ce6). In a previous own study we showed that aPDT using Ce6 and TB in combination with VIS + wIRA efficiently eradicated initial and mature oral biofilms [4]. In that study the killing effects were demonstrated by live/dead-staining and the determination of CFU. On the other hand, the present study dealt with an aspect of the aPDT which has not been investigated, so far. It revealed the alteration in composition of aPDT treated biofilms not only by their quantification but also by a comprehensive identification of the surviving oral microorganisms. Due to the fact that oral biofilm formation is a result of an ecological balance between different microorganisms living in the same environment, the present study revealed that such a balance can be destroyed by the aPDT.

As discussed in earlier studies [3,4], VIS + wIRA is generated by a halogen lamp, with a continuous spectrum of light in the range of 570–1400 nm. After water filtration the harmful infrared B and C wavelengths were decreased, allowing the infrared A component, which penetrates tissues with a low thermal load, to remain. Due to its proven wound healing, pain alleviation and inflammation preventing effects [18,35], aPDT using VIS + wIRA could be a promising therapy adjuvant in treating biofilm associated oral diseases, including periimplantitis and periodontitis. Moreover, the VIS + wIRA light source was found to be less painful than only VIS, and safe when applied with treatment doses of up to 30 min [16]. These treatment times are much higher than the light doses used in the present report (5 min). Indeed, application of aPDT using VIS + wIRA combined with the photosensitizers TB and Ce6 was found to have high antimicrobial effects on planktonic cultures of representative pathogenic oral bacteria, as well as on the initial and mature oral biofilms [3,4], which is in agreement with the results presented in this report.

The evaluation of the antimicrobial effects of aPDT on planktonic oral bacteria can only serve as a preliminary test which indicates a proof of principle for the chosen aPDT parameters such as photosensitizers, light dose and applied energy. In medicine, biofilms represent an never-ending source of infection, since microorganisms which are organized in biofilms are up to 1000 times more resistant against antimicrobial agents such as disinfectants and antibiotics than planktonic cells [6]. Moreover, single-species or multiple-species biofilms formed *in vitro* cannot sufficiently reflect the complex situation in the oral cavity where the high diversity of salivary components included in the salivary pellicle provides the basis for the anchor of the initial oral biofilm [11,25,36]. Furthermore, dental plaque biofilm consists of up to 700 different microbial species [37,38], making it impossible to simulate this situation *in vitro*. Finally, the extracellular matrix of *in situ* biofilm contains a variety of polymeric substances such as carbohydrates, nucleic acids, residues of bacterial components and extracellular enzymes [8]. Each of these components could interact with the photosensitizers, which have different chemical structures and origins. TB, for example is a cationic phenothiazinium-based photodynamic molecule which directly binds to negatively-charged lipopolysaccharide (LPS) sites on the outer cell membranes as well as to the peptidoglycan surfaces of Gram-positive bacteria [39–41]. Ce6, the second photosensitizer tested in this study is a chlorophyll “a”-based second-generation photosensitizing agent with a structural resemblance to porphyrins [42]. Using the live/dead staining technique we were able to show in one of our earlier studies that Ce6 can better permeate the mature oral biofilm formed *in situ* [4]. Keeping this in mind we used a splint system which can be loaded with bovine enamel slabs as a reliable substitute substratum for human enamel, and which we have successfully used in previous studies to obtain initial and mature oral biofilm for *ex vivo* experiments [4,11,14]. To the best of our knowledge, the effects of aPDT on the bacterial composition of native *in situ* oral biofilm have not been studied yet.

The oral biofilm consisting of hundreds of different bacterial species is formed by a reproducible and concerted coaggregation pattern [43–45]. In addition to coaggregation, metabolic interaction and cell-cell signaling are important processes which contribute to a functionally active oral biofilm in which the different bacterial species create the protective environment necessary for the stability of the biofilm [44]. For example, *Fusobacterium nucleatum* was found to interact as a bridging species between early and late colonizers during the process of biofilm formation [46,47]. Interestingly, the results of the present study showed that this species could not be detected in the biofilm after the application of aPDT using TB. Another important group in oral biofilms consists of oral streptococci which are the early colonizers of the biofilm. This group was highly reduced (14% compared to 32% in the untreated biofim) in the mature oral biofilm by Ce6-mediated aPDT. Changes such as these in microbial biofilm composition suggest high alteration effects of TB- and Ce6-mediated aPDT on the microbial composition of dental plaque biofilm. Beighton [48] described the oral biofilm as an ecological system which may be beneficially altered to reduce biofilm associated diseases such as caries and periodontitis. This underlines that destroying the balance within the oral biofilm community could be an alternative approach to therapy. The author emphasized that besides diet, targeted alteration of the bacterial composition of the biofilm should be kept in mind as a method to affect its ecological balance. The differences in biofilm penetration patterns of both tested photosensitisers and the light source could have played an important role for the survival of microorganisms after the application of aPDT. In a recent own study, we showed that the deepest layers of the oral biofilm were not affected by the aPDT [4]. Due to the different chemical structures of the photosensitizers, their interaction with the extracellular polymeric substances (EPS) of the oral biofilm could not be excluded. These interactions could inhibit the penetration of photosensitisers into the deeper layers of the biofilm. It should also be taken into consideration that the survival of microorganisms with different chemical structure of the microbial cell envelope could correlate with their varied sensitivity towards the tested photosensitizers. The interindividual discrepancies in the detected oral microorganisms reflect the age and gender differences as well as different eating habits within the participants of the study.

The heterogeneous survival pattern for the aPDT-treated oral microorganisms revealed an individual response profile of the participants to aPDT. This could correlate with the individual composition of saliva and the existing oral microbiota prior to aPDT. Especially the individual survival pattern of oral microorganisms should be taken into consideration due to the fact that oral microbiota coexist in interactions with each other and the salivary components in the oral cavity. In addition, with regard to the development of periodontitis it has been shown that the bacterial composition of biofilm as well as shifts in its composition are associated with periodontal health status [49,50]. All in all, changing the bacterial composition of the oral biofilm influences oral health status.

Our results showed that not only was the bacterial load of initial and mature biofilm significantly reduced by aPDT using VIS + wIRA, but also that the composition of surviving bacteria was highly altered. As expected, these effects were greater on the initial biofilm compared to 3-day-old oral biofilm. The spectrum of the detected bacteria in the oral cavity can vary among the different individuals. In the present study we have detected 20 different species in the untreated initial adhesion, which comprises of mainly Gram-positive bacterial species (early colonizers). The fact that hundreds of bacterial species have identified in the oral cavity describes the salivary bacteria as well as members of the oral supragingival and subgingival biofilm. Additionally, the identification of a high bacterial diversity correlates with the detection methods, namely cultural and molecular techniques. In the present study, only the culture technique was applied, since only the spectrum of surviving cultivable bacteria was studied.

Considering that some natural photosensitizers may be selectively effective against only some Gram-positive or Gram-negative bacteria, studying their effects on oral biofilm ecology should be a focus of future studies. The question now arises as to how such an altered biofilm develops in the oral cavity if the volunteers again wear the splint systems loaded with the previously treated biofilm. Another interesting aspect for research is to examine how many times the mature oral biofilm should be treated by aPDT to eradicate all pathogenic species with regard to caries, periodontitis and periimplantitis.

In conclusion, the present study has shown that aPDT using VIS + wIRA combined with the photosensitizers TB or Ce6 has remarkable potential not only in the eradication of initial and mature oral biofilm, but also in dramatically altering the surviving remaining biofilm. The results of the present report encourage the clinical use of aPDT with VIS + wIRA for periimplantitis and periodontitis treatment.

## Supporting Information

S1 Table Raw data depicting the values of the colony forming units (CFUs) after antimicrobial photodynamic treatment (aPDT). The CFUs were determined in the phases of initial adhesion and biofilm formation.
(XLSX)

S2 Table Raw data depicting the values of the colony forming units (CFUs) for each of the identified bacterial species after antimicrobial photodynamic treatment (aPDT). For aPDT, the photosensitizers toluidine blue (TB) and chlorine e6 (Ce6) were used to treat the initially adhered oral microorganisms. 0.2% Chlorhexidine (CHX) served as a positive control, while untreated initial biofilms were used as negative controls. The Log values as well as the bacterial diversity of the biofilms are also demonstrated on the tables.
(XLSX)

S3 Table Raw data depicting the values of the colony forming units (CFUs) for each of the identified bacterial species after antimicrobial photodynamic treatment (aPDT). For aPDT, the photosensitizers toluidine blue (TB) and chlorine e6 (Ce6) were used to treat mature oral biofilms. 0.2% Chlorhexidine (CHX) served as a positive control, while untreated mature biofilms were used as negative controls. The Log values as well as the bacterial diversity of the biofilms are also demonstrated on the tables.
(XLSX)
